# Assessing the Validity and Impact of Remote Digital Image Reading in Fungal Diagnostics

**DOI:** 10.1007/s11046-025-01012-x

**Published:** 2025-11-07

**Authors:** Vilhelmina Lundgren, Özlem Dogan, Anna Ekwall-Larson, Christine Stenström, Erja Chryssanthou, Maria Guglielmeti, Ylva Närström, Patrik Dinnétz, Silvia Botero-Kleiven, Volkan Özenci

**Affiliations:** 1 Division of Clinical Microbiology, Department of Laboratory Medicine, Karolinska Institutet, Huddinge, Sweden; 2 Department of Clinical Microbiology, Karolinska University Hospital, SE 141 86 Huddinge, Stockholm, Sweden; 3 Department of Clinical Microbiology, Karolinska University Hospital, Solna, Sweden; 4 School of Natural Sciences, Technology and Environmental Studies, Södertörn University, Huddinge, Sweden; 5 Department of Medical Microbiology, School of Medicine, Koc University, Istanbul, Turkey

**Keywords:** Clinical mycology, Invasive fungal infections, Digital diagnostics, Telemycology

## Abstract

Mycological diagnostics play a crucial role in patient management and treatment of invasive fungal infections. Despite the significant global burden of fungal diseases, awareness and diagnostic capabilities in mycology laboratories lag behind other microbiological disciplines. Mycological diagnostics often require microscopic analysis of clinical samples and culture. The interpretation of microscopy requires extensive expertise in clinical mycology. This study aimed to explore the feasibility of remote digital reading for preliminary identification of fungi. In this study, five mycology-trained participants were asked to analyze a total of 474 images divided into three main groups of yeasts (73 images), filamentous fungi (341 images), and direct fluorescent microscopy from clinical samples (60 images). The accuracy of the assessments varied, with an average correct decision rate between 78 and 93% across the three image groups. Individual participant’s performance showed a mean accuracy rate ranging between 76 and 92%. A significant difference was observed in the assessment accuracy across specimen groups and among individual participants (p < 0.05). However, there was no significant interaction effect between participants and image group (p = 0.118). In conclusion, telemycology offers a promising alternative to standard microscopy diagnostics of fungal infections, especially in settings where skilled mycologists are lacking, including low- and middle-income countries.

## Introduction

Fungal infections represent a significant and growing challenge worldwide, particularly affecting immunocompromised individuals such as those with HIV, leukemia, or organ transplant recipients [1]. These infections range from superficial skin conditions to life-threatening systemic diseases. The recently launched Fungal Priority Pathogen List by the WHO aims to strengthen global research and policy efforts against fungal infections, enhancing diagnostic capabilities and clinical mycology training worldwide while identifying key areas for targeted action to bolster the global response to fungal infections [2]. The increasing numbers of invasive fungal infections (IFIs) in different groups of susceptible patients requires solid competence in fungal diagnostics including morphological diagnosis [3]. Despite the high global incidence and the severity of fungal infections, diagnostic capabilities in many regions, especially in low- and middle-income countries, are inadequate [4].

The traditional methods for diagnosing fungal infections involve evaluation of clinical samples with direct microscopy and various culture techniques, requiring highly trained personnel. These methods are often unavailable in regions with limited medical infrastructure, leading to underdiagnosis of IFIs and delayed treatment. Recently it was reported that only five out of 164 contacted institutions in Africa fulfilled the minimum laboratory requirements for becoming a blue status fungal centre by the European Confederation of Medical Mycology (ECMM) [5]. In clinical microbiology, fungal diagnostics have traditionally relied on labor-intensive and resource-demanding methods. Most institutions use morphological identification methods, that, while effective, require experienced clinical staff. A recent survey showed that manual microscopy still is the diagnostic method of choice for 75% of European institutions [6]. Although conventional methods form the foundation of clinical mycology diagnosis in many centers worldwide, there is a shortage of trained and experienced laboratory personnel and specialists. Considering that clinical mycology training is extremely challenging, and recruiting medical professionals who work 24/7 in this field is nearly impossible for most centers, standardized remote digital image reading could enable specialists working in different time zones worldwide to provide remote diagnostic support to distant collaborating laboratories. Therefore, implementing a world-wide network using remote diagnosis in clinical mycology can enable specialists in any laboratory to provide services in regions with limited expertise around the clock. This might lead to timely accurate diagnoses of fungal infections, and immediate start with efficient treatment.

The integration of automation and digital technologies in clinical microbiology has been transformative in bacterial diagnostics but has scarcely been implemented in clinical mycology. Modern advancements include automated plate inoculation, incubation, and digital plate reading, which have improved workflow efficiency and reduced the time to diagnosis of bacterial infections [7]. These tools have the potential to revolutionize fungal diagnostics as well as providing faster and more accurate identification methods. Although artificial intelligence, particularly deep learning and convolutional neural networks, is already enhancing image analysis in other medical fields and shows promise for application in fungal diagnostics, these systems are still in development, and it will take time for a universally accessible and validated system to emerge [8]. In contrast, currently there are tools for collecting high quality images and sharing those with experts across different geographical regions. However, there is no information about the performance of remote digital analysis in clinical mycology. The aim of the present study was to develop and validate the remote digital image reading for fungal diagnostics.

## Material and Methods

### Specimen Collection and Identification of Fungi

To assess the feasibility of remote digital reading of clinical mycology samples, a library of macroscopic and microscopic images of fungal specimens was established. All specimens used in this study were isolated from clinical samples at the Karolinska University Laboratory, Department of Microbiology. For species identification, standard examination of morphological features, Matrix-assisted laser desorption ionization-time of flight mass spectrometry (MALDI-TOF MS) and agglutination assays were used as reference techniques. Subcultures of the isolated fungi were inoculated on Chrom/Dex agar for yeasts which is two-sided petri dish with one side including chromogenic medium (CHROMagar Candida Plus, CHROMagar, Paris, France) and the other side including dextrose agar with 5% horse blood, vancomycin, and chloramphenicol (Condolab, Madrid, Spain) and potato dextrose agar for filamentous fungi. Cultures were incubated at 37 °C for three days for yeasts and at 30 °C for 4–7 days for filamentous fungi, until adequate growth was observed. Yeast colony colour was assessed according to instructions of the manufacturer.

Species included in the study were selected based on their abundance in the clinical setting. Each species was represented by five specimens whenever possible (Table 1). In total five of each *Candida albicans, C. dubliniensis, C. glabrata, C. tropicalis, C. parapsilosis, Clavispora lusitaniae* and *Pichia kudriavzevii* were included. Additionally*, C. auris* was included with three isolates. For filamentous fungi, five isolates of *Aspergillus fumigatus, A. flavus, A. niger, A. terreus*, *Mucor spp*., *Rhizopus spp*., *Fusarium spp., Scedosporium spp., Paecilomyces* spp., *Penicillium* spp. and two *Lichtheimia* spp were included.

**Table 1:** List of species collected for digital imaging, and the number of specimens included in the digital reading experiment

Yeasts	No. of samples	Filamentous fungi	No. of samples
*Candida albicans*	5	*Aspergillus fumigatus*	5
*Candida dubliniensis*	5	*Aspergillus flavus*	5
*Candida glabrata*	5	*Aspergillus niger*	5
*Candida tropicalis*	5	*Aspergillus terreus*	5
*Candida parapsilosis*	5	*Mucor* spp.	5
*Candida auris*	3	*Rhizopus* spp.	5
*Clavispora lusitaniae*	5	*Lichtheimia* spp.	2
*Pichia kudriavzevii*	5	*Fusarium* spp.	5
		*Scedosporium* spp.	5
		*Paecilomyces* spp.	5
		*Penicillium* spp.	5

### Configuration of the Image Library

Digital images were categorized into three groups: (i) direct fluorescence microscopy of clinical samples; (ii) filamentous fungi (macroscopic and light microscopy images from colonies); and (iii) yeast (macroscopic images from colonies).

#### Direct Fluorescence Microscopy of Clinical Samples

**Fig. 1:**
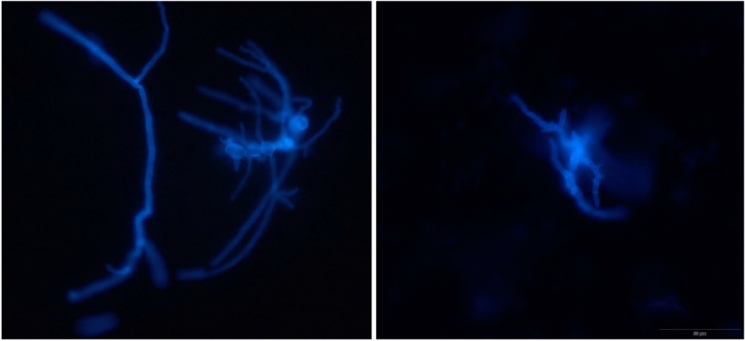
Filamentous fungi from clinical samples in direct fluorescence microscopy (20 × objective). The images show clusters of filamentous fungal hyphae

Fluorescence microscopy was conducted on sample materials deposited and dried on glass slides and stained with 0.04% Blankophor® within 2% NaOH. This process involved capturing images of yeast cells, hyphae, and artifacts using objectives with magnifications of 10x, 20x, and 40x, resulting in a total of 20 images per category. The sample materials comprised various patient-derived specimens including cell pellets from drainage fluids, bronchoalveolar lavage (BAL), sputum and tissue biopsies (Fig. 1). The imaging was performed using an Olympus BX51 microscope, which was equipped with a pE-300lite COOLED white light source and an Olympus SC50 camera, ensuring high-quality fluorescence imaging across all samples.

#### Light Microscopy of Filamentous Fungi

**Fig. 2:**
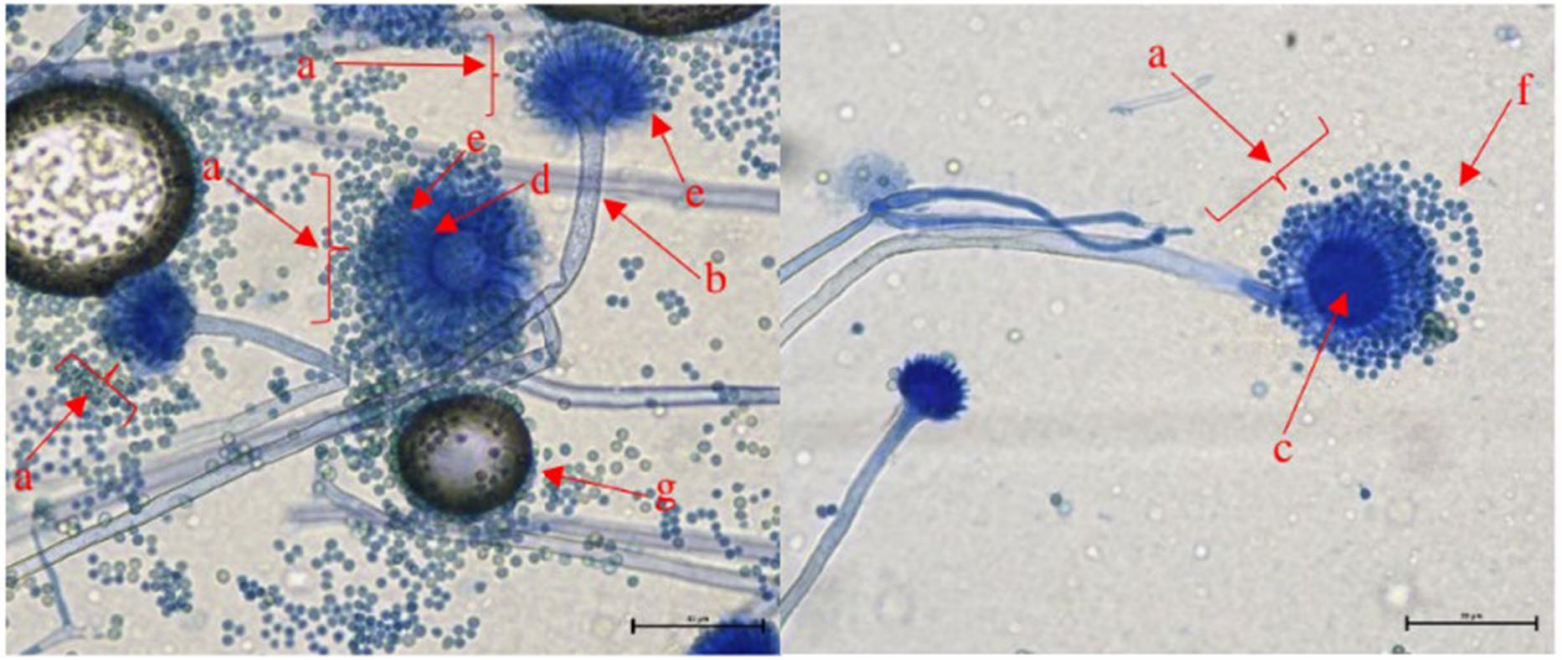
Light microscopy images of *Aspergillus flavus* prepared from culture (40 × objective). The images show characteristic morphological structures of the *Aspergillus flavus* conidiophores **a** including roughened stalks **b** a round vesicle **c** metulae **d** phialides **e** and round spores **f** Dark circles **g** in the left image show air bubbles formed during the preparation of the tape mount

For light microscopy of filamentous fungi, 4–6 images were taken of Scotch tape preparation mounts stained with 0.1% methylene blue in lactic acid. Each filamentous fungus was photographed to highlight spores and asexual reproductive structures in accordance with the described characteristic morphology of the species (Fig. 2). A Nikon Eclipse Ci microscope equipped with a camera (The Imaging Source, model DFK 33UX264) was utilized, with standardized light microscopy settings for filamentous fungi. Exposure time was set to automatic. The condenser aperture diaphragm was individually adjusted for each objective lens in relation to the specific microscopy slide, facilitating sufficiently illuminated images. Objective lenses magnification of 20 × and 40 × were used for all isolates. In the image library corresponding macroscopic and microscopic images for individual specimens were grouped together to facilitate comparative analysis.

#### Macroscopic Images

Macroscopic images of the fungal cultures were captured using a Fujifilm X-T200 digital camera under standardized conditions to ensure consistency across all images. The camera was positioned at a fixed height of 11.5 cm above the agar plates. Exposure and focus settings were automated, while brightness was manually adjusted for each plate to optimize image quality.

**Fig. 3:**
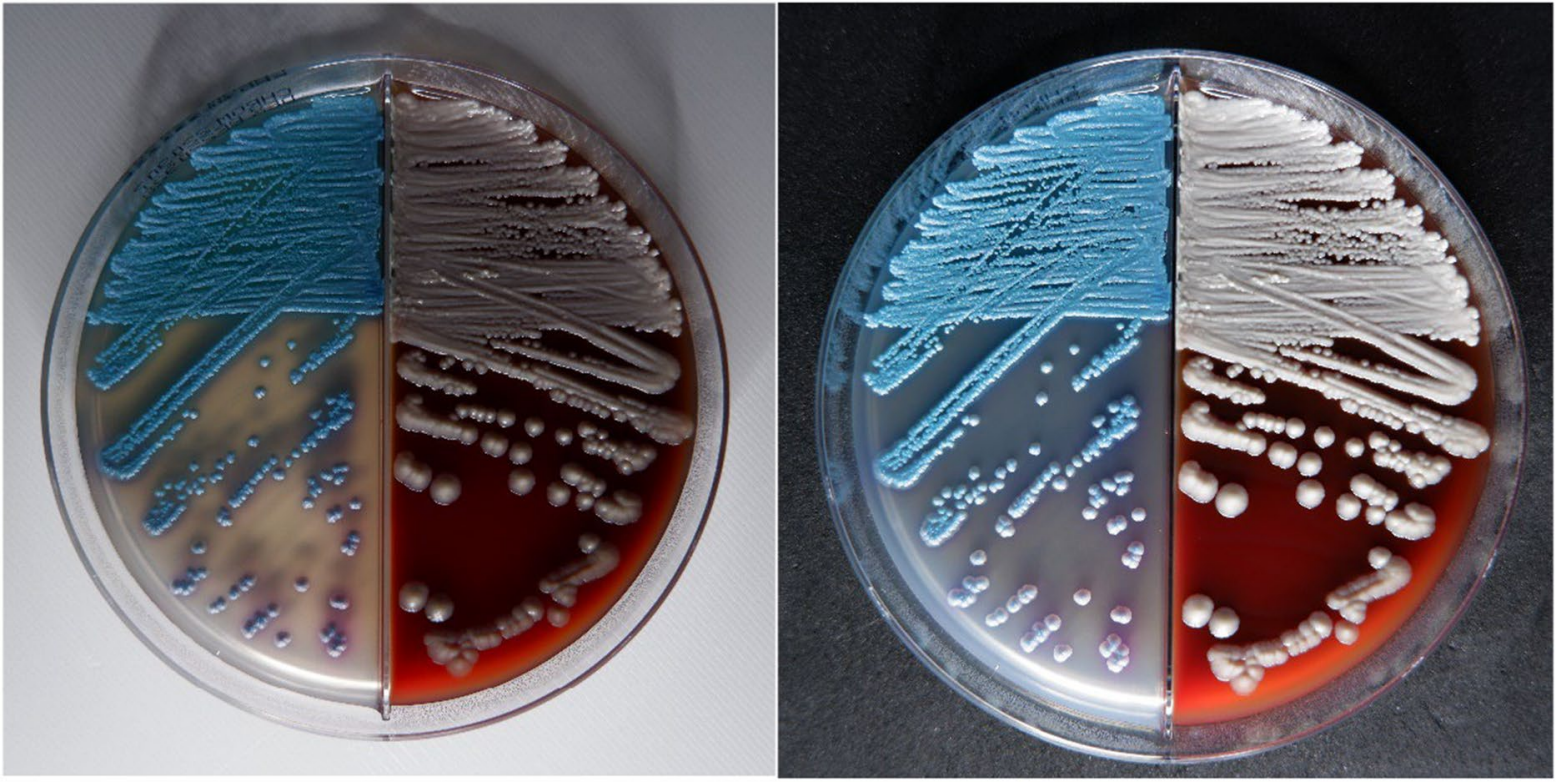
Macroscopic Image of *Candida tropicalis* on Chrom/DEX agar. Front side, white and black background respectively

**Fig. 4:**
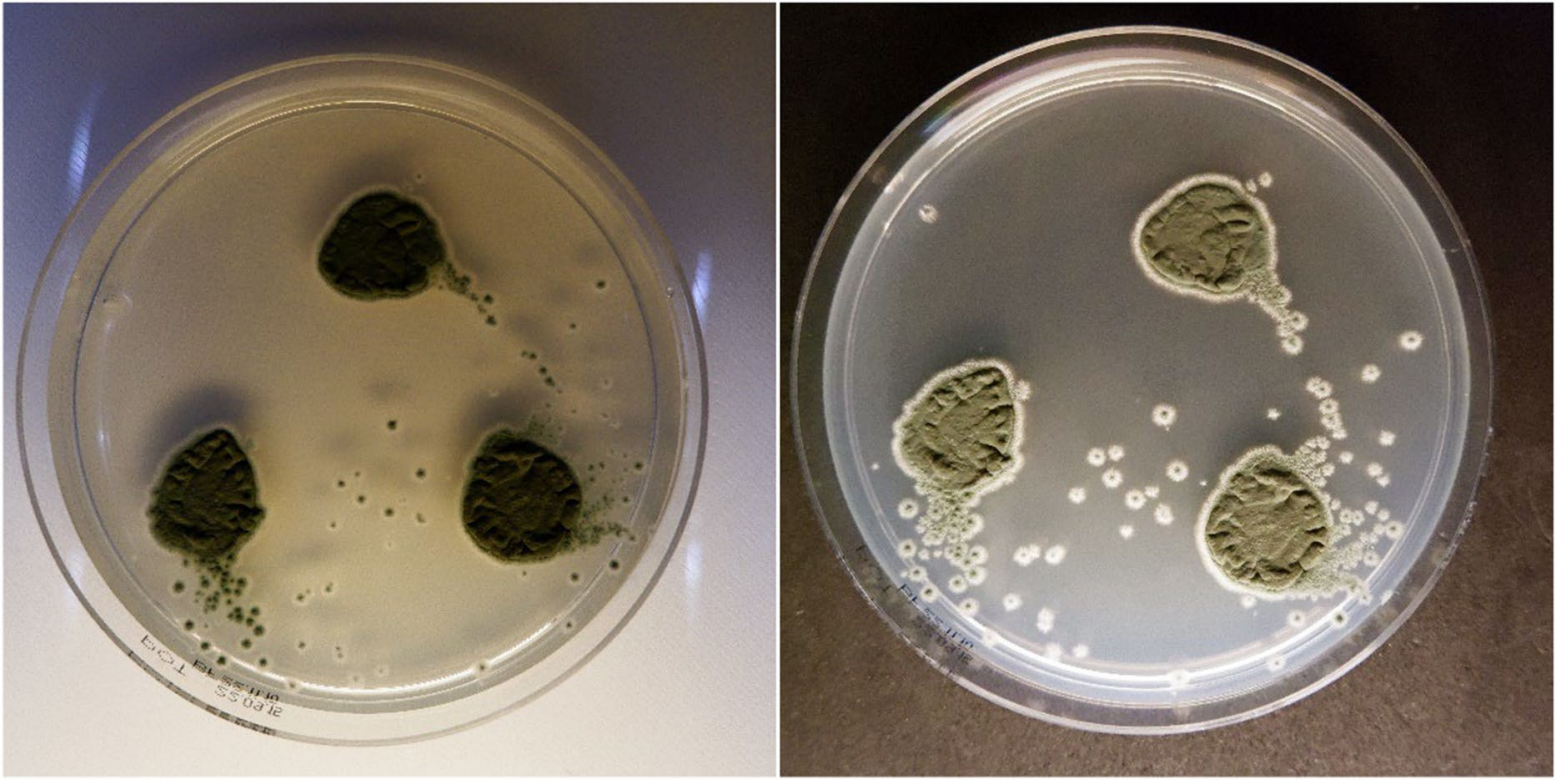
Macroscopic Image of *Penicillium* species on potato dextrose agar. Front side, white and black background respectively

During the photography process, white and black background colors were tested to optimize image contrast of colonies on agar plates. For yeasts, both backgrounds were used consistently in the same image, allowing microbiologists to evaluate colony color against two contrasting backgrounds (Fig. 3). Generally, the white background provided the most realistic contrast for observing yeast colonies. Conversely, the black background enhanced the contrast between the filamentous fungi and their backdrop, making fungal structures more distinguishable. Based on these observations, all images of filamentous fungi were subsequently captured against a black background to ensure better visibility and differentiation of features (Fig. 4).

### Blind Reading of the Digital Images

Five staff members from Karolinska University Laboratory with experience in mycological diagnostics ranging between 2 and 35 years participated in a blind reading test of digital images to evaluate the feasibility of digital reading of the configured library. Two of the participants had three years or less experience, while one had 20 years and another 25 years of professional background. The remaining participant had been working in the field of microbiology for 35 years. Based on the educational backgrounds, four of the 5 participants are biomedical analysts, while one was a clinical microbiologist. The participants were allotted 16 days to complete the blind assessment and to submit their responses. For direct microscopy from clinical sample evaluations (Fig. 1), the participants had to select among four options provided as; yeast, filamentous fungi, artifacts/non-fungal structure, and not able to decide. For macroscopic yeast images, participants recorded the perceived color of colonies following recommendations from the CHROMagar manufacturer and laboratory-established color interpretations (Table 2). For filamentous fungi, species- or, when not possible, genus-level identification was required.

**Table 2:** Categories of yeast grouped by colony colour

Species	Colony colour according on CHROMagar Plus
*Pichia kudriavzevii, Candida parapsilosis, Candida glabrata, Clavispora lusitaniae*	Shades of pink/ white/ purple
*Candida albicans, Candida dubliniensis*	Shades of green
*Candida tropicalis*	Shades of blue
*Candida auris*	Shades of white with blue diffusion in agar

### Data Collection and Statistical Analyses

All specimens were identified using standard assessment methods, allowing each participant’s specific assessment from the digital images to be judged as correct or incorrect. This design enabled calculations of proportion of accuracy with 95% confidence intervals (CIs), for each participant, for different types of specimens in the three digital image groups. The binary responses [correct, incorrect] for all participants were analyzed using a generalized linear model (glm) with binomial error distribution and logit link. The full model included the binomial answers [correct, incorrect] as dependent variable, the image group [direct microscopy, filamentous fungi, yeast], participant, and the two-way interaction between image group and participant as independent variables. This allowed testing whether variation in accuracy differed among participants and if differences were consistent among the different image groups. The interaction between image group and participant was non-significant and was dropped from the final model, that only included the main effects. Significance was determined using likelihood ratio (LR) tests followed by multiple comparisons among all levels in each main effect using Tukey contrasts adjusting all p values to the family wide significance level of α = 0.05. All statistical analyses were conducted in R 4.2.1 [9] and RStudio 2022.07.1. [10] Effect plots were generated using packages ggeffects [11] and ggplot2 [12].

## Results

### Sample Distribution

In this study, participants were asked to analyze an image library configured of a total of 474 images. These images were divided into three main groups as yeasts, filamentous fungi and direct microscopy. For all yeast species except *C. auris*, both front and back side images were taken, totaling 70 images (2 images each for 35 species). However, for the three *C. auris* samples, only one front image was taken. Thus, a total of 73 images related to yeasts were included. For the evaluation of filamentous fungi, both macroscopic and microscopic (stained with lactophenol cotton blue) images were taken for all 52 filamentous fungi isolates. A total of 341 filamentous fungi images were included in the study, consisting of 156 macroscopic (including front and back sides) and 185 microscopic images. Finally, 60 direct microscopy clinical sample images stained with fluorescent dye were included in the study.

### Interpretation of the blind reading analysis

#### Performance in digital image group

**Fig. 5:**
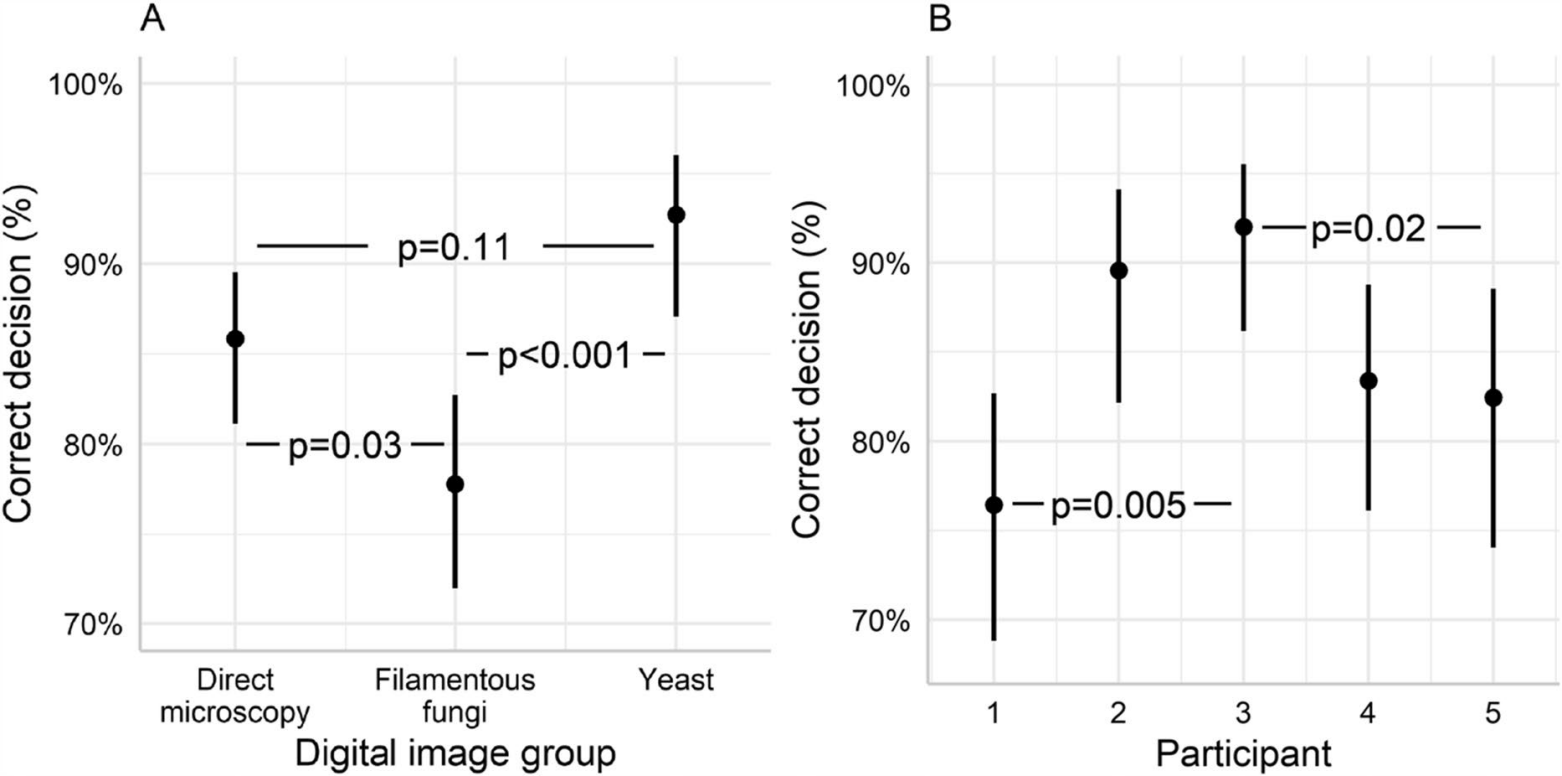
**A** Average correct decision rate (%) in the blind reading test for all five participants for each image group. Error bars denote 95% CI: direct microscopy 86% (0.81–0.90), filamentous fungi 78% (0.72–0.83), and yeast 93% (0.87–0.96). All p-values for pairwise comparisons indicted by horizontal lines show adjusted Tukey contrasts. **B** Average correct decision rate for individual participants in the blind reading test. Error bars denote 95% CI. Significant pairwise comparisons between participant 1 and 3, and 3 and 5, show adjusted Tukey contrasts. All other pairwise contrasts were non-significant (p > 0.05)

The analysis of the blind reading experiment showed a significant main effect of image group on interpretation success (LR = 17.46, df = 2, p < 0.001, glm). The correct decision rate for all participants was significantly higher for yeast (p < 0.001) and direct microscopy (p = 0.03) compared to filamentous fungi (Fig. 5A).

#### Overall performance of individual participants

There was also a significant effect of the main factor participant on the decision success for the different image groups (LR = 17.74, df = 4, p = 0.001, glm). Using Tukey’s post hoc test, we showed that the significant differences among participants were due to participant 3 having a significantly higher success rate compared to participant 1 and 5 (Fig. 5B). All other pairwise comparisons among participants were non-significant (Fig. 5B).

#### Performance of individual participants in different image group

**Fig. 6:**
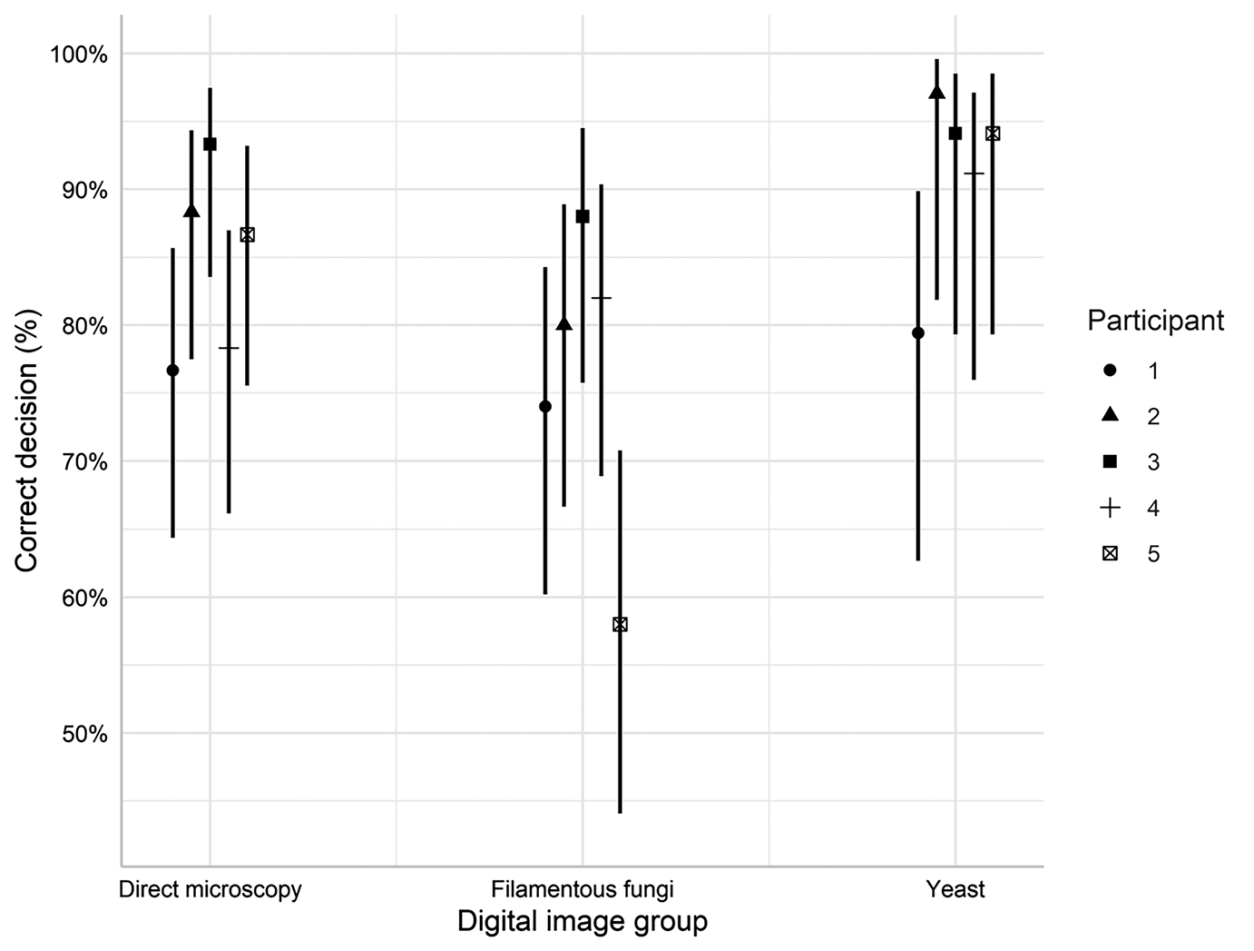
Correct decision rate (%) for individual participant for each image group. Error bars indicate 95% CI. The interaction between participant and digital image group as non-significant (p = 0.118, glm)

There was no significant interaction between image group and participant (LR = 12.84, df = 8, p = 0.118, glm). (Fig. 6) However, looking at each participant’s success rate for all different image groups we can see that the significant differences between participant 3, and participant 1 and 5 (Fig. 5B), have different causes (Table 3). Participant 1 has similar but low success rate for all three specimen types, while participant 5 shows large variation with high success rate for direct microscopy and yeast, but very low for filamentous fungi (Fig. 6). All other participants show high consistency among image groups (Fig. 6).

**Table 3:** Correct decision rates of each participant for different image groups

Image group	Correct decision rate % (min–max)				
	Participant				
	1	2	3	4	5
Direct microscopy	77%	88%	93%	78%	87%
	(0.64–0.86)	(0.77–0.94)	(0.84–0.97)	(0.66–0.87)	(0.76–0.93)
Filamentous fungi	74%	80%	88%	82%	58%
	(0.60–0.84)	(0.67–0.89)	(0.76–0.95)	(0.69–0.90)	(0.44–0.71)
Yeast	79%	97%	94%	91%	94%
	(0.63–0.90)	(0.82–1.00)	(0.79–0.99)	(0.76–0.97)	(0.79–0.99)

## Discussion

This study explored the feasibility of remote digital image reading for fungal diagnostics in clinical mycology. Participants independently analyzed a diverse set of digital images including yeasts, filamentous fungi, and direct microscopic images of clinical samples. The study concluded that remote digital diagnosis has the potential to enhance fungal diagnostic practices, especially in regions with limited access to skilled microbiologists, by providing timely and accurate diagnostic support remotely.

The digital remote reading method achieved an overall concordance rate of 78–93% with standard mycological diagnostic techniques. Analysis by image group revealed significantly lower identification accuracy for filamentous fungi compared to yeast and direct microscopy. The underlying reasons for lower accuracy (78%) in the assessment of some of the images is not known. It is possible that the limited numbers of images per specimen or the current study’s digital method limitation in not allowing users to scroll through images, unlike manual microscopy might play a role. For direct mycological diagnosis of filamentous fungi, identifying key features like dichotomous branching and septa formation are crucial but challenging with two-dimensional images only. This limitation in assessing image depth could explain the lower identification score for filamentous fungi. Identifying rarer species such as those in the order Mucorales requires extensive mycological experience and detailed examination. Thus, to achieve more consistent identification rates in direct microscopy, inclusion of more images and/or whole slide imaging techniques might be implemented in remote digital diagnosis in future.

This study involved participants with varied education and experience backgrounds which probably affected the difference among their overall correct decision performance. In another study, researchers evaluated interlaboratory differences in Gram-stained smear evaluation and found significant variations between evaluation scores and emphasized the urgent need for a standardized automated slide reading method [13]. We observed that apart from one participant’s filamentous fungi evaluation score, all participants scored over 74%. The results obtained in the present study are promising and indicating that the present method can be useful for daily clinical practice. Furthermore, storing these image libraries and using them as remote mycology education and training modules could represent a new approach in training, especially considering that today’s knowledge transfer in mycology predominantly relies on on-site microscopic and macroscopic evaluation.

In this study, the highest concordance rate (93%) was achieved with yeast identification by using chromogenic media. Although chromogenic media primarily provide preliminary identification, they can be crucial for selecting effective antifungal treatments which can significantly impact mortality rates. Additionally, some chromogenic media can now provide rapid preliminary identification of the emerging pathogen *C. auris*, which is now becoming a global concern.

In a recent study, researchers established a diagnostic network hub in a middle-low income country to improve the diagnosis of fungal infections. This hub facilitated progress in diagnosing fungal infections, which in turn increased awareness among clinicians about these infections. As a result, the mortality rate associated with invasive fungal infections decreased by 7% [14]. Although digital image reading and remote diagnosis technologies are still in their infancy today, with the impending contributions of the artificial intelligence and machine learning era, these tools could soon connect laboratories worldwide. This would be especially beneficial in middle- and low-income countries, where limited resources have made basic mycological diagnostics exceedingly difficult [15]. Consequently, this advancement could significantly enhance the timely diagnosis of fungal infections, which are becoming an increasingly severe global issue. By directly boosting clinical awareness, these technologies could also have a meaningful impact on reducing mortality rates associated with fungal infections.

Addressing the challenges of personnel education and coordination, the integration of machine learning and artificial intelligence (AI) is increasingly becoming a valuable tool in medical diagnostics, helping to streamline processes and improve diagnostic accuracy. AI-based medical diagnosis has recently been launched and implemented in radiology and pathology, showing promising results in both accuracy and time reduction in these fields. While automated laboratory inoculation and culturing systems in clinical microbiology laboratories are more commonly available nowadays, implementation of digital reading and AI-based diagnostic tools are limited [16]. Koo et al. [17] performed a study with a neural network detecting hyphae in superficial fungal infections. With 838 images in 100 × and 40 × magnification, sensitivity and specificity were reported as 93.2% and 89%, respectively. In another study, researchers aimed to implement a machine learning-based image analysis scheme to support dermatologists in the diagnosis of fungal hypha in clinical samples. They configured a test dataset and found the sensitivity for hyphae was 94% and 89% for singular and clustered hyphae, respectively, with a mean exclusion rate of 91% for false positive objects [18]. Fisher et al. [19] compared an automated slide reading system with manual microscopy and according to culture results, the specificity was higher (90.8%) for automated digital imaging compared with manual microscopy (87.7%). It is evident that AI-based interpretation of fungal samples certainly will be developed and take place in the clinical routine. However, the implementation of reliable AI-based diagnostics, ethical aspects and the cost of these methods are still major challenges for using them in the near future. The present method, with remote analysis of images is safe and easy to implement in the clinical routine. Moreover, configuring education modules from digital image libraries is not only useful for remote mycology education but also for machine learning baselines. Collaboration between laboratories based on the present method will be another advantage of implementing remote digital analysis for fungal infections.

There are several limitations in the present study. The number and diversity of isolates included are limited. However, the species analyzed in this study cover common pathogens encountered in daily mycology practice. The capacity and technical knowledge of computer usage ability may have affected the success rate of each participant. In this study, we did not use a preliminary information document; a detailed guideline will be provided for upcoming telemycology studies. This study was only conducted in a single center. To address this issue, a multi-center study involving different institutions from different countries is being designed. Finally, this study used retrospectively collected samples and did not assess the clinical efficacy or impact of remote digital diagnosis on real-time clinical practice. Broader studies incorporating real-time reading and clinical outcome data are needed to demonstrate the possible effect of remote digital reading on disease outcome.

This study has several strengths. Firstly, it is the first study in the field of clinical mycology to comparatively evaluate the compatibility of remote digital reading with conventional methods. Additionally, the participants involved in this study were selected from diverse educational and experience backgrounds, contributing to meaningful diversity. Another strength of the study is that it includes a clinically representative variety of microorganisms and clinical samples, thereby encompassing many areas where direct microscopy is utilized in clinical mycology.

## Conclusions

This study shows the potential application of remote digital reading in clinical mycology. The present results demonstrate that remote digital reading yields results comparable to conventional methods. However, the variability of the results in diagnostic accuracy across different sample types emphasizes the need for further refinement of these technologies. Future multi-center studies are warranted to show that the method can potentially be used in collaboration between distant laboratories.
