# Correction: Assessing the Validity and Impact of Remote Digital Image Reading in Fungal Diagnostics

**DOI:** 10.1007/s11046-025-01049-y

**Published:** 2026-01-13

**Authors:** Vilhelmina Lundgren, Özlem Dogan, Anna Ekwall-Larson, Christine Stenström, Erja Chryssanthou, Maria Guglielmeti, Ylva Närström, Patrik Dinnétz, Silvia Botero-Kleiven, Volkan Özenci

**Affiliations:** 1 Division of Clinical Microbiology, Department of Laboratory Medicine, Karolinska Institutet, Huddinge, Sweden; 2 Department of Medical Microbiology, School of Medicine, Koc University, Istanbul, Turkey; 3 Department of Clinical Microbiology, Karolinska University Hospital, Solna, Sweden; 4 Department of Clinical Microbiology, Karolinska University Hospital, SE 141 86 Huddinge, Stockholm, Sweden; 5 School of Natural Sciences, Technology and Environmental Studies, Södertörn University, Huddinge, Sweden

**Correction to: Mycopathologia (2025) 190:114** 10.1007/s11046-025-01012-x

In the original publication of this article, Fig. 6 appeared in an incomplete form. This has now been corrected in the online publication. For completeness and transparency, the correct and old incorrect versions are displayed below.

The original article has been corrected.

Incorrect Fig. 6

**Fig. 6:**
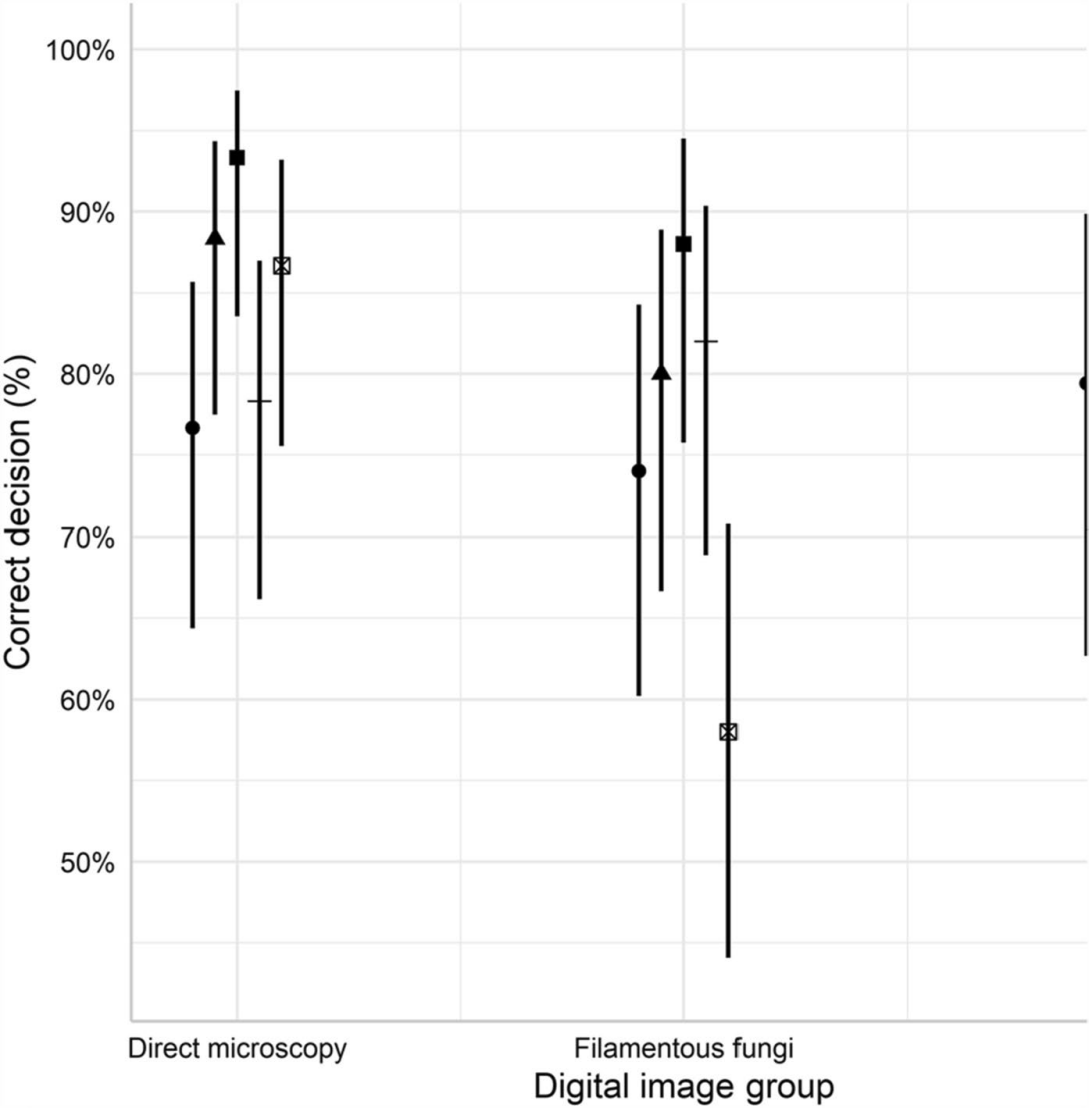
Correct decision rate (%) for individual participant for each image group. Error bars indicate 95% CI. The interaction between participant and digital image group as non-significant (p = 0.118, glm)

Correct Fig. 6

**Fig. 6:**
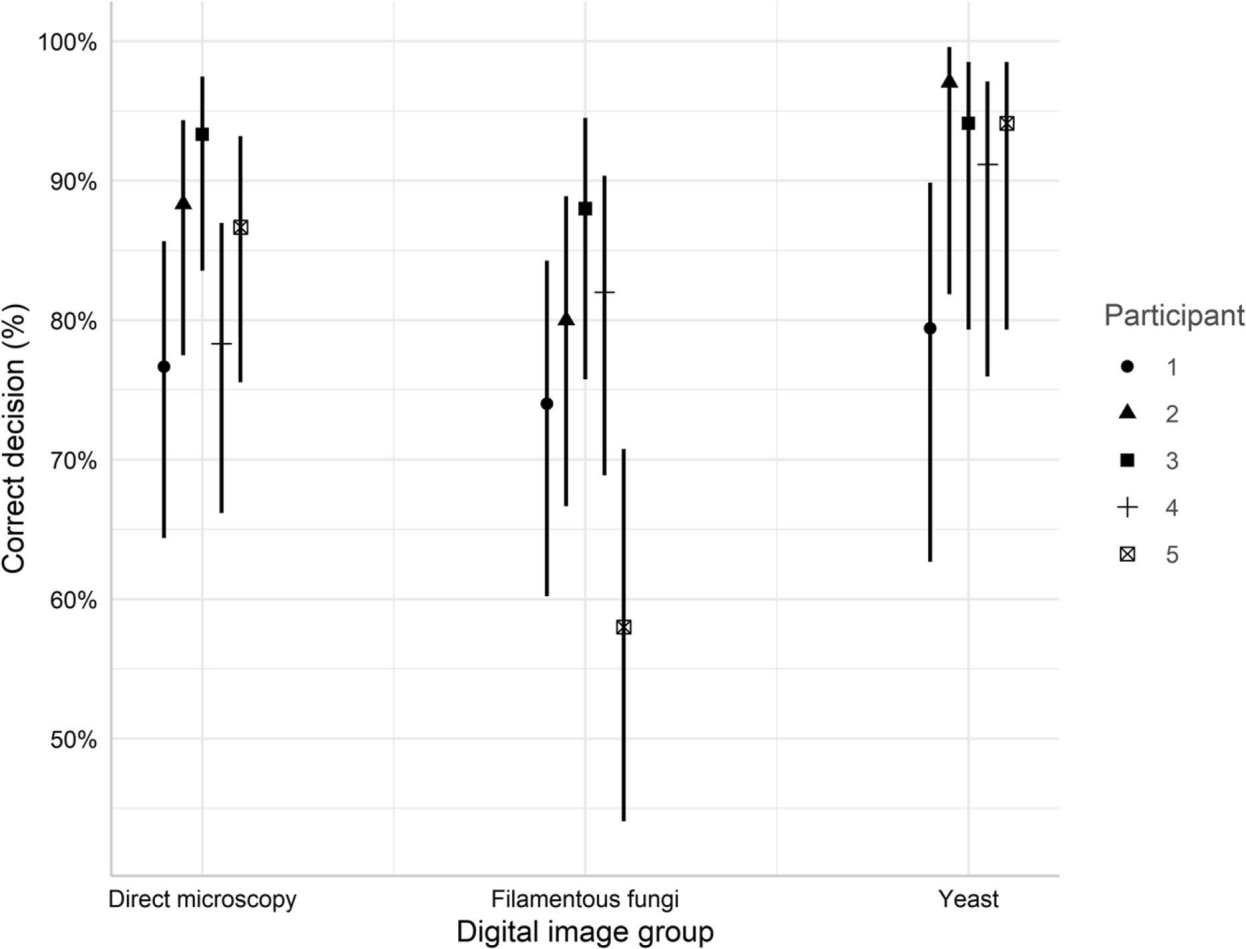
Correct decision rate (%) for individual participant for each image group. Error bars indicate 95% CI. The interaction between participant and digital image group as non-significant (p = 0.118, glm)

